# Developing and evaluating a localized health education curriculum in Qinghai, China: a cluster randomized experimental study in high schools

**DOI:** 10.3389/fpubh.2026.1867706

**Published:** 2026-08-17

**Authors:** Leyi Cao, Yueting Zhang, Bing Shi

**Affiliations:** 1 School of Physical Education, Qinghai Normal University, Xining, China; 2 Physical Education School, Shaanxi Normal University, Xi'an, China

**Keywords:** adolescent health literacy, China, cluster randomized study, health education, local curriculum, Qinghai

## Abstract

**Background:**

This study aimed to develop and preliminarily evaluate a localized health education curriculum tailored to the cultural, environmental, and health needs of high school students in Qinghai Province, China.

**Methods:**

A pretest-posttest pilot cluster-randomized design was employed involving 180 Grade 11 students from four intact classes. Within both the ethnic minority and general education categories, one class was randomly assigned to the experimental group and the other to the control group. The primary outcome was the post-test overall mean health literacy score adjusted for the corresponding pre-test score. A fixed-effects model including intervention group, class type, and their interaction was used. A linear mixed model with a random intercept for class was additionally conducted as a sensitivity analysis.

**Results:**

The adjusted mean scores were 3.774 for the experimental group and 3.514 for the control group, yielding an adjusted mean difference of 0.259 [*SE* = 0.008, 95% CI (0.243, 0.275), *F* (1, 175) = 1034.856, *p* < 0.001, partial η^2^ =0.855]. The interaction between intervention group and class type was not statistically significant (*p* = 0.553). The differences in mean change between the experimental and corresponding control classes were 0.259 in the ethnic minority category and 0.263 in the general education category. Exploratory analyses across the seven dimensions of health literacy also showed greater improvements in the experimental group.

**Conclusion:**

The findings provide preliminary evidence that a localized health education curriculum may improve health literacy among high school students in Qinghai Province. However, because only four clusters were included, class-level variance could not be estimated reliably and cluster-level confounding cannot be ruled out. The findings should therefore be interpreted as preliminary rather than definitive evidence of curriculum effectiveness.

## 1 Introduction

Health literacy has become a critical determinant of public health and individual wellbeing, particularly for adolescents, who are at a formative stage of developing lifelong health behaviors and attitudes (1, 2). The World Health Organization (WHO) emphasizes the essential role of school-based health education in promoting equitable access to health information and the acquisition of health competencies, especially in regions marked by socioeconomic and cultural disparities. Numerous international frameworks advocate for context-sensitive and culturally relevant health education as an effective strategy to reduce health inequities and improve population health outcomes. In China, recent policy reforms have sought to more effectively integrate health education into the national school curriculum. The General High School Physical Education and Health Curriculum Standards (2017 Edition) identify health literacy as a core component of students' holistic development.

However, the implementation of health education remains uneven across regions, with particularly notable disparities in underdeveloped western provinces such as Qinghai. Qinghai is characterized by its high-altitude environment, multiethnic population—including Tibetan, Hui, and Mongolian communities—and distinctive health risks such as endemic infectious diseases and lifestyle-related conditions. These contextual factors render the standardized national curriculum insufficient to fully meet local health education needs. Consequently, developing an effective localized health education curriculum that aligns with the region's environmental and cultural characteristics has become an important direction for promoting adolescent health literacy and reducing regional disparities in health education. However, empirical evidence on how localized and culturally responsive health education curricula can effectively enhance adolescent health literacy in multiethnic and underdeveloped regions such as Qinghai remains limited. This study addresses this gap by developing and evaluating a localized health education curriculum tailored to the province's unique ecological and cultural context.

### 1.1 Literature review

In the field of health education, as well as in curriculum development and implementation, countries such as the United States, Japan, South Korea, and the United Kingdom have accumulated extensive research findings and practical experience. These international cases provide valuable insights for the theoretical advancement and practical improvement of health education not only in China but also across Asia and the world.

#### 1.1.1 Development of localized health education in the United States

In the United States, physical and health education serves as a core subject aimed at cultivating wellness knowledge and social competence (3). The governance of health education is highly decentralized; while the federal government provides broad policy frameworks, authority over curriculum design is vested in state agencies and school districts. This structure facilitates localized curriculum development, ensuring content aligns with students' specific regional needs (4). A defining characteristic of this model is extensive stakeholder participation. As noted by Jean (5), involving diverse participants, ranging from teachers and administrators to community members, in the design process guarantees the richness and adaptability of regional health education.

#### 1.1.2 Development of localized health education in Japan and South Korea

East Asian models typically balance centralized standards with local flexibility. According to Liu (6), in Japan, the “Health and Physical Education Model” integrates health instruction with physical activity. While national standards ensure uniformity, local schools retain the autonomy to adapt curricula to their specific contexts. Similarly, South Korea incorporates health education into an integrated curriculum. Although the central government mandates core content, schools can adapt elective modules based on local conditions and the specific needs of teachers and students (7), reflecting a trend toward localized practice within a national system.

#### 1.1.3 Development of localized health education in European Countries

The United Kingdom employs a national framework that supports localized implementation, exemplified by the Personal, Social, and Health Education (PSHE) program (8). This approach emphasizes the holistic development of students' moral and social dimensions (9). Programs such as the Social and Emotional Aspects of Learning (SEAL) operate under national guidance but are flexibly adapted at the school level to address regional needs (10). In contrast, health education in Spain faces challenges due to ambiguous curricular foundations (11, 12). Research indicates that without effective school-based localization, students often rely on external sources for health information, and adolescent needs remain frequently neglected by standard educational approaches (13).

#### 1.1.4 Development of localized health education in Australia

Australia adopts a three-tiered governance model involving federal, state, and school levels (14, 15). The federal government sets general principles, while state governments oversee implementation, allowing for significant contextual adaptability (14). This decentralized structure is particularly crucial for addressing the specific needs of diverse populations. For instance, Susan and Singh (16) emphasized that designing curricula tailored to the sociocultural contexts of Indigenous communities is essential for effective health promotion. Such initiatives illustrate how localized practice can operate effectively within a national framework to support equity.

#### 1.1.5 Empirical evidence on the effectiveness of localized health education

While policy frameworks provide the structural basis for localization, a growing body of empirical research supports the effectiveness of localized and place-based educational strategies in improving student outcomes. Theoretically, place-based education (PBE) emphasizes the meaningful integration of learning with local physical, cultural, and social environments. Semken et al. (17) demonstrated that curricula grounded in local contexts significantly enhance students' sense of place and content mastery. Similarly, Sianturi et al. (18) found that localized curricula designed for Indigenous communities improved academic engagement while strengthening teachers' cultural responsiveness, thereby reducing educational disparities among minority students.

In the specific domain of health promotion, empirical studies have reported consistent benefits of school-based and culturally adapted interventions. A systematic review by Kirchhoff and Keller (19) further indicated that effective school-based life-skills education should be age-appropriate, connected to real-life situations, and provide sustained opportunities for students to apply acquired skills in everyday contexts. Similarly, Khanal et al. (20) emphasized the importance of structured school-based health literacy interventions for developing adolescents' functional health competencies. Recent randomized controlled trials (RCTs) provide robust evidence for this approach. For instance, Yüksek and Ayaz-Alkaya (21) found that health literacy education significantly improved scores among early adolescents in a controlled setting. Similarly, Akca and Ayaz-Alkaya (22) demonstrated that targeted interventions could effectively modify nutrition and exercise behaviors. Further evidence by Fung et al. (23) indicates that culturally responsive interventions are particularly effective for the psychosocial wellbeing of ethnic minority youth. Collectively, these studies suggest that effective localized health education interventions typically share several core features, including alignment with local health risks, integration of cultural knowledge, and responsiveness to students' lived experiences.

Despite these positive findings, the application of empirically validated localized health education models in China remains uneven. Although scholars like Cairang cuo and Wanma cuo (24) have long argued for the “strong vitality” of school-based curriculum development in Qinghai's ethnic schools, their work was largely qualitative case studies. Consistent with earlier observations by Begoray and Banister (25), the lack of a coherent theoretical and evaluative framework has limited the systematic assessment of many health education programs. Moreover, existing health education research in China is largely concentrated in urban and economically developed eastern regions, with comparatively limited empirical attention to rural and ethnic minority communities (26, 27). As a result, rigorous cluster randomized experimental studies examining comprehensive localized health education curricula in high-altitude, multiethnic, and socioeconomically underdeveloped regions, such as Qinghai Province, remain scarce. The present study addresses this gap by developing and empirically evaluating a localized health education curriculum that integrates regional ecological characteristics with national health literacy standards.

## 2 Methods

### 2.1 Research design

This study adopted a pretest-posttest control group cluster randomized experimental design, using classes as the unit of allocation, to preliminarily evaluate the implementation effects of a localized health education curriculum compared with the current national standardized curriculum. The intervention was conducted over one academic semester (approximately 3.5 months) at a public high school in Haibei Prefecture, Qinghai Province. This region was selected as the research site due to its distinct high-altitude environment, multiethnic culture, and local health issues, making it an appropriate setting to examine the alignment between a regionally tailored curriculum and specific contextual factors.

The study examined both the temporal dimension (pre-test and post-test) and the group dimension (experimental group and control group). Given that each class category included only one experimental class and one control class, this study is positioned as a preliminary, exploratory evaluation. While the pre- and post-test results can be used to compare change patterns across different classes, they cannot completely disentangle the effects of the curriculum intervention from the inherent characteristics of the classes; therefore, causal interpretations should be made with caution.

### 2.2 Participants

A total of 180 Grade 11 students from four intact classes were included in this study, with classes serving as the unit of cluster random allocation. The final sample included 66 male students (36.7%) and 114 female students (63.3%). The sample comprised 99 students from ethnic minority classes and 81 students from general education classes. The ethnic minority classes consisted of over 95% minority students, primarily Tibetan, Hui, and Mongolian; the general education classes were predominantly composed of Han students, with a small number of ethnic minority students. The experimental and control groups in the ethnic minority category consisted of 55 and 44 students, respectively, while those in the general education category consisted of 39 and 42 students, respectively.

Within both the ethnic minority and general education categories, two intact classes with similar academic performance and grade levels were selected as eligible clusters and then assigned to either the experimental or control group using a simple random procedure. Specifically, an independent researcher not involved in the intervention delivery entered the class identifiers into a computer-generated random number sequence. Within each category, one class received the localized health education curriculum, while the other class continued to study the national standard curriculum.

To minimize variations caused by teacher factors, both the experimental and control classes within each class category were taught by the same health education teacher. Furthermore, both groups were kept consistent in terms of class schedules, duration of single sessions, and assessment procedures. This arrangement helped control for teacher-level differences within the same class category. However, because there was only one experimental class and one control class per category, cluster-level confounding factors such as teacher characteristics, peer interactions, and class environments could not be completely ruled out. The sample consisted of the four eligible intact Grade 11 classes available at the participating school; therefore, no *a priori* sample-size calculation for a cluster-randomized trial was conducted. Given the inclusion of only four clusters, the study was treated as a preliminary evaluation rather than a confirmatory trial designed to achieve a prespecified level of statistical power. The basic characteristics of the four classes are presented in Table 1.

**Table 1:** Sample composition and pre-/post-test overall health literacy scores of the four clusters (classes).

Class ID	Class type	Group assignment	Teacher	*n*	Pre-test (*M* ±*SD*)	Post-test (*M* ±*SD*)	Mean change
1	Ethnic minority	Experimental	Teacher A	55	3.257 ± 0.329	3.541 ± 0.288	0.284
2	Ethnic minority	Control	Teacher A	44	3.219 ± 0.308	3.244 ± 0.288	0.025
3	General	Experimental	Teacher B	39	3.753 ± 0.313	4.041 ± 0.234	0.288
4	General	Control	Teacher B	42	3.813 ± 0.265	3.838 ± 0.249	0.025

The experimental and control classes in the ethnic minority category were taught by the same teacher; the experimental and control classes in the general education category were taught by another teacher. Mean Change = Post-test mean—Pre-test mean.

### 2.3 Instrumentation

To assess students' health literacy levels, a customized self-assessment questionnaire was developed for this study based on three sources: (1) the General High School Physical Education and Health Curriculum Standards (2017 Edition) issued by China's Ministry of Education; (2) major public health challenges and priority health issues specific to Qinghai Province; and (3) findings from semi-structured interviews with experts in health education and public health. The finalized questionnaire comprised 42 items organized into seven thematic domains: (1) Basic health knowledge and skills; (2) Nutrition and food safety; (3) Hygiene and disease prevention; (4) Environment, health, and physical activity; (5) Safe exercise and injury management; (6) Emergency response and safety awareness; (7) Psychological, moral, and social adaptation. All items were rated on a five-point Likert scale, with higher scores indicating greater self-perceived health literacy.

The questionnaire was developed through a systematic and evidence-based process to ensure both content validity and reliability. Initially, the Health Literacy Self-Assessment Questionnaire for High School Students in Qinghai Province was drafted based on curriculum standards, local environmental characteristics, students' health literacy profiles, and expert feedback. The preliminary version contained 43 multiple-choice items across seven thematic domains. To refine the instrument, the Delphi method was employed in two rounds of expert consultation involving seven specialists in health education, curriculum design, and public health. Experts provided feedback both online and offline. During the first Delphi round, experts identified several issues, including incomplete coverage of health dimensions, minor logical inconsistencies, redundant or ambiguously phrased items, and partial misalignment between some items and their intended domains. The research team revised and optimized the questionnaire accordingly, resulting in a refined version with 42 items more closely aligned with the official curriculum framework.

In the second Delphi round, the same seven experts rated the importance of each domain and item on a five-level scale: “very important” (9 points), “important” (7 points), “moderately important” (5 points), “less important” (3 points), and “unimportant” (1 point). Statistical analysis indicated high consensus among the experts: the mean importance score for all seven domains exceeded 7.0, and the coefficient of variation (CV) for each domain was below 0.15. As shown in Table 2, a similar pattern was observed for all 42 items, which also achieved mean importance ratings above 7.0 and CV values below 0.15. This indicates that the items are representative of their corresponding domains and effectively reflect the multidimensional structure of students' health literacy.

**Table 2:** Statistical parameters of expert ratings for each thematic domain.

Thematic domain	Standard deviation	Mean value	Coefficient of variation
A	0.000	9.000	0.000
B	1.069	7.857	0.136
C	0.7559	8.714	0.087
D	0.000	9.000	0.000
E	0.000	9.000	0.000
F	0.976	8.429	0.116
G	0.000	9.000	0.000

A pilot test was subsequently conducted with one Grade 11 class to examine the internal consistency and reliability of the instrument. Data analysis using IBM SPSS Statistics for Windows, Version 20.0 (IBM Corp., Armonk, NY, United States). revealed that all seven thematic domains demonstrated satisfactory internal consistency, with Cronbach's α coefficients above 0.70, indicating that the questionnaire items were coherent and stable within each construct, as detailed in Table 3. Taken together, the finalized 42-item questionnaire exhibited good content validity, expert consensus, and internal reliability, making it a psychometrically sound tool for assessing high school students' health literacy in the Qinghai regional context.

**Table 3:** Reliability statistics for each thematic domain of the health education questionnaire.

Health education themes	Cronbach's alpha
Basic health knowledge and skills	0.745
Nutrition and food safety	0.719
Hygiene and disease prevention	0.755
Environmental health and physical activity	0.774
Safe Exercise and the Prevention and Management of Common Sports Injuries	0.801
Emergency Response, Safety Awareness, and Risk Prevention Competency	0.726
Mental Health and Social Adaptation Ability	0.732

### 2.4 Curriculum intervention

The experimental curriculum systematically integrated region-specific and culturally relevant content into the framework of the national high school physical education and health curriculum standards. These localized adaptations primarily addressed Qinghai Province's endemic disease prevention, health risks associated with high-altitude environments, and traditional health beliefs and practices among ethnic minority groups.

To enhance implementation consistency, two teachers, responsible for the ethnic minority and general education classes, respectively, underwent a 5-day specialized training program prior to implementation. The training covered research objectives, curriculum framework, localized instructional content, communication and pedagogical strategies, cultural sensitivity, and the control of teacher-related variables. Due to the nature of classroom-based curriculum interventions, it was not possible to blind teachers and students to the group assignments.

During the intervention phase, teachers in the experimental group selected and delivered 3–5 thematic units from the localized curriculum within regular health education class hours. The control group, in contrast, continued to follow the standard national curriculum and was not exposed to the newly developed localized content or related instructional materials. Both the experimental and control groups maintained procedural equivalence by following the same weekly class schedule, session duration, and assessment procedures.

Implementation consistency was supported through standardized teacher training, a common curriculum framework, shared instructional materials, aligned schedules, and common classroom implementation requirements. However, no standardized fidelity instrument, independent classroom observation, formal adherence checklist, or prospectively defined quantitative fidelity threshold was used; therefore, formal quantitative intervention fidelity could not be established.

### 2.5 Outcomes

The single primary outcome of this study was defined as the post-intervention overall mean score of health literacy, adjusted for the pre-intervention overall mean score. The post-test scores across the seven thematic dimensions of health literacy served as secondary outcomes to explore the specific content areas potentially affected by the curriculum. Given the multiplicity of secondary outcomes, their results are interpreted solely in an exploratory manner.

### 2.6 Data collection and analysis

Data were collected before and after the intervention using the health literacy questionnaire described in Section 2.3. The questionnaire was administered uniformly in classrooms under the supervision of homeroom and physical education teachers to ensure standardization and procedural guidance. Students were informed that participation was voluntary, responses were anonymous, data would be used strictly for research purposes, and their participation or answers would not affect their academic performance or school evaluations.

First, the sample size, pre-test mean, post-test mean, and average change were reported for each of the four classes. For the primary outcome analysis, a fixed-effects model was employed using the post-test overall mean score of health literacy as the dependent variable and the pre-test overall mean score as the covariate. The intervention group, class type, and the interaction term of “intervention group × class type” were included in the model, and Type III sum of squares tests were used. Estimated marginal means were calculated to report the adjusted means for the experimental and control groups, the adjusted mean difference, standard error (*SE*), 95% confidence intervals (CI), and partial η^2^. The estimated marginal means were calculated at the overall pre-test mean and averaged across the two class types with equal weighting.

To examine the sensitivity of the results to the handling of the cluster structure, a linear mixed model with a random intercept for class was additionally fitted. Given that there were only four clusters, the class-level random variance might not be stably estimated; therefore, this model served exclusively as a sensitivity analysis rather than conclusive evidence that class-level confounding was completely eliminated. The seven secondary outcomes were analyzed using the same baseline-adjusted model, with Holm correction applied to the *p*-values of the main intervention effects. The adjusted group differences, 95% CIs, and partial η^2^ were also reported. All tests were two-tailed, with a significance level set at 0.05.

### 2.7 Ethics and blinding

The study was reviewed and approved by the Academic Ethics Committee of Qinghai Normal University on July 25, 2023, before data collection began. Because this was a classroom-based educational intervention, neither teachers nor students could be blinded to group allocation. Questionnaire administration was conducted by personnel who were aware of the class assignments, and no formal blinding was implemented during data entry or statistical analysis. Before participation, legal guardians were informed about the study and provided verbal informed consent, while students provided verbal assent. Participation was voluntary, and students were informed that participation or withdrawal would not affect their academic performance or school evaluation. Questionnaires were collected anonymously, and all data were kept confidential and used solely for research purposes.

## 3 Results

### 3.1 Descriptive results at the sample and class levels

All 180 students included in the analysis had complete pre-test and post-test data for overall health literacy. As shown in Table 1, the mean changes for the experimental and control classes in the ethnic minority category were 0.284 and 0.025, respectively, resulting in a difference in mean change of 0.259. For the general education category, the mean changes for the experimental and control classes were 0.288 and 0.025, respectively, with a difference in mean change of 0.263. The direction and magnitude of these changes were largely consistent across both class types. The pre-test overall mean scores for the experimental and control classes in the ethnic minority category were 3.257 and 3.219, respectively, while those for the general education category were 3.753 and 3.813, respectively. Given that each condition corresponds to only one class, tests for baseline differences cannot be considered conclusive evidence of complete equivalence at the cluster level.

### 3.2 Primary outcome: baseline-adjusted overall health literacy

After controlling for pre-test overall health literacy, the main effect of the intervention group was statistically significant (*F* (1, 175) = 1034.856, *p* < 0.001, partial η^2^ = 0.855). The main effect of class type was also statistically significant (*F* (1, 175) = 53.457, *p* < 0.001, partial η^2^ = 0.234). The interaction between the intervention group and class type was not significant (*F* (1, 175) = 0.353, *p* = 0.553, partial η^2^ = 0.002).

As presented in Table 4, after calculating at the overall pre-test mean and averaging equally across class types, the adjusted mean for the experimental group was 3.774 (95% CI: 3.763–3.785), and for the control group, it was 3.514 (95% CI: 3.503–3.526). The adjusted mean difference between the experimental and control groups was 0.259 (SE = 0.008, 95% CI: 0.243–0.275). The direction of this difference is consistent with the class-level change results observed in both class types.

**Table 4:** Estimated marginal means and intervention effects for the primary outcome.

Parameter	Estimate	*SE*	95% CI	*F*	*p*	Partial η^2^
Adjusted mean for control group	3.514	0.006	3.503–3.526	—	—	—
Adjusted mean for experimental group	3.774	0.006	3.763–3.785	—	—	—
Adjusted mean difference (Experimental—Control)	0.259	0.008	0.243–0.275	1,034.856	<0.001	0.855

The dependent variable is the post-test overall mean score of health literacy; the covariate is the pre-test overall mean score. The model includes the intervention group, class type, and their interaction term. Estimated marginal means are calculated at the overall pre-test mean of 3.485 and averaged across the two class types with equal weighting. Partial η^2^ represents the effect size from the individual-level fixed-effects model.

In the linear mixed model sensitivity analysis, the estimation of the class random intercept variance reached the parameter boundary, resulting in a non-positive definite Hessian matrix warning. This result should not be interpreted as the absence of a class effect; rather, it indicates that with only four clusters, the between-class variance could not be stably estimated. Therefore, the standard errors, confidence intervals, and partial η^2^ presented in Table 4 primarily reflect the results of the individual-level model and may underestimate the uncertainty at the cluster level.

### 3.3 Secondary outcomes: thematic dimensions of health literacy

The baseline-adjusted results for the seven thematic dimensions are presented in Table 5. The adjusted differences between the experimental and control groups were all positive, with Holm-corrected *p*-values not exceeding 0.002 across all dimensions. The adjusted differences for each dimension ranged from 0.158 to 0.413. The “intervention group × class type” interaction was statistically significant for the Basic Health Knowledge and Skills dimension (*p* < 0.001): the adjusted intervention difference was 0.500 (95% CI: 0.449–0.550) in the ethnic minority class category and 0.327 (95% CI: 0.272–0.382) in the general education class category. The interaction terms for the remaining six dimensions did not reach statistical significance.

**Table 5:** Baseline-adjusted intervention effects across the seven health literacy dimensions.

Health literacy dimension	Adjusted difference	*SE*	95% CI	Holm-corrected *p*	Partial η^2^
Basic health knowledge and skills	0.413	0.019	0.376–0.450	<0.001	0.732
Nutrition and food safety	0.255	0.017	0.221–0.289	<0.001	0.558
Hygiene and disease prevention	0.300	0.018	0.265–0.335	<0.001	0.619
Environment, health, and physical activity	0.158	0.051	0.057–0.258	0.002	0.052
Safe exercise and injury management	0.239	0.017	0.206–0.272	<0.001	0.539
Emergency response and safety awareness	0.217	0.013	0.190–0.243	<0.001	0.599
Psychological health and social adaptation	0.198	0.015	0.169–0.228	<0.001	0.503

Adjusted difference = adjusted mean of the experimental group—adjusted mean of the control group. Each model controlled for the pre-test score of the corresponding dimension and included class type and the “intervention group × class type” interaction term. Secondary outcomes are exploratory analyses; partial η^2^ is also based on individual-level fixed-effects models.

Regarding the unadjusted changes at the class level, the mean increase in the Basic Health Knowledge and Skills dimension was 0.524 for the ethnic minority experimental class and 0.344 for the general education experimental class, whereas the corresponding control classes only increased by 0.027 and 0.029, respectively. Across the other dimensions, the mean increases in the experimental classes were generally higher than those in the control classes. These results suggest that the curriculum's effects may simultaneously encompass knowledge, behavioral, and psychosocial domains. However, because the measurement instrument was developed based on the curriculum standards and localized content, there might be some overlap between the scale items and the curriculum content. Therefore, strong causal interpretations should not be drawn from these secondary outcomes.

## 4 Discussion

This study conducted a preliminary evaluation of a localized health education curriculum for high school students in Qinghai Province. Baseline-adjusted analyses revealed that the post-test overall health literacy of the experimental group was higher than that of the control group, and the direction of this change was largely consistent across both ethnic minority and general education classes. These results align with recent international empirical evidence. For instance, a randomized controlled trial by Yüksek and Ayaz-Alkaya (21) confirmed that health literacy education significantly elevates scores in early adolescence. More broadly, Kirchhoff and Keller (19) emphasize that school-based life-skills education should be age-appropriate, engage students with real-life situations, and provide sustained opportunities to apply skills in everyday contexts. However, it is important to emphasize that the large effect sizes observed in our study were derived from individual-level fixed-effects models, which cannot substitute for adequate cluster-level adjustments, nor should they be interpreted as definitive proof of the curriculum's effectiveness. Therefore, the observed intervention effects should be interpreted as preliminary rather than definitive estimates of curriculum effectiveness.

Linking curriculum content to Qinghai Province's high-altitude environment, endemic health risks, and multiethnic culture likely enhanced the comprehensibility and applicability of health knowledge. This finding supports the theoretical framework of Place-Based Education (PBE), which emphasizes embedding learning content within local natural, cultural, and social environments (17). From the perspective of Bronfenbrenner's (28) ecological systems theory, the localized curriculum effectively optimized the “microsystem” of the school environment. By making the educational content congruent with the students' “macrosystem” (i.e., their regional and cultural background), the intervention fostered a more supportive learning ecology. Unlike fluid sociocultural variables, the natural environment remains relatively stable over time, providing a consistent dimension for shaping ecological health literacy.

An important observed pattern was that the intervention classes showed greater improvements across both the general education and ethnic minority categories. This mirrors the findings of Sianturi et al. (18), who demonstrated that culturally responsive curricula could bridge educational gaps for minority students by validating their cultural identities. Incorporating traditional health beliefs of Tibetan and Hui communities likely increased the curriculum's legitimacy and resonance. Furthermore, while Fung et al. (23) focused specifically on mindfulness interventions, their conclusion that culturally adapted strategies are vital for the psychosocial wellbeing of ethnic minority youth is corroborated by our results in the “Psychological Health and Social Adaptation” domain.

The magnitude of the effects varied across different dimensions. Regarding specific behavioral domains, our results are broadly consistent with those of Akca and Ayaz-Alkaya (22), whose study suggested that targeted education may contribute to changes in nutrition and exercise behaviors. However, the adjusted difference in the “Environment, Health, and Physical Activity” dimension was relatively small, whereas knowledge-based dimensions exhibited larger differences. This suggests that while knowledge can be acquired rapidly within a single semester, the internalization of deep-seated behaviors and psychosocial adaptation requires prolonged exposure, practice, and environmental reinforcement.

The study has several potential implications. First, the observed findings suggest that regionally adaptive curriculum models warrant further evaluation alongside uniform national curricula. This aligns with recent insights by Yang et al. (29), who emphasized that addressing regional imbalances in student health requires adjusting the focus of education to local conditions. Second, it highlights the necessity of teacher training in cultural responsiveness to ensure faithful implementation. Fundamentally, health education is a shared social responsibility involving families and communities (30). Although the intervention classes showed greater improvements than the corresponding control classes, future research should explore how school-based education can be connected with community health practices.

Several limitations should be acknowledged. First, although allocation occurred at the class level, the study included only four intact classes, with one intervention class and one control class within each educational category. Consequently, class-level variance could not be estimated reliably, and the intervention effect cannot be fully separated from class-specific influences, including teacher characteristics, peer interactions, and classroom environment. The standard errors, confidence intervals, and effect sizes obtained from the individual-level fixed-effects models may therefore underestimate uncertainty. Second, teachers and students could not be blinded to group allocation because of the classroom-based nature of the intervention, and no independent blinded outcome assessment was conducted. Third, the self-developed questionnaire was based partly on the curriculum standards and localized intervention content, which may have increased alignment between the intervention and the measured outcomes. The seven domain-specific analyses should therefore be interpreted as exploratory. Fourth, the intervention lasted only one semester, and the study was conducted in a single school, limiting assessment of long-term effects and generalizability to other schools or regions. Future studies should include substantially more schools and classes, prespecify primary outcomes, use independently validated or less intervention-aligned measures, incorporate longer follow-up periods, and prospectively assess intervention fidelity.

## 5 Conclusion

In this study, the experimental classes demonstrated a greater magnitude of improvement in health literacy than the corresponding control classes, and the direction of this effect was generally consistent across both ethnic minority and general education classes. These findings provide preliminary evidence that a culturally and regionally adapted health education curriculum in Qinghai Province may yield positive effects on adolescent health literacy. However, because only four clusters were included, class-level confounding cannot be completely ruled out, and the current results should not be used to draw definitive causal inferences. Future cluster randomized studies with larger numbers of schools and classes, properly accounting for intra-class correlations, and incorporating longer follow-up periods are needed to further validate these findings.

## Data Availability

The datasets generated and/or analyzed during the current study are not publicly available because they contain sensitive information involving adolescent and ethnic minority participants. The datasets are available from the corresponding author upon reasonable request and subject to appropriate ethical approval.
